# Telmisartan/hydrochlorothiazide versus valsartan/hydrochlorothiazide in obese hypertensive patients with type 2 diabetes: the SMOOTH study

**DOI:** 10.1186/1475-2840-6-28

**Published:** 2007-10-02

**Authors:** Arya M Sharma, Jaime Davidson, Stephen Koval, Yves Lacourcière

**Affiliations:** 1 McMaster University, Hamilton, ON, Canada; 2 The Endocrine and Diabetes Associates of Texas, Dallas, TX, USA; 3 Boehringer Ingelheim Pharmaceuticals, Inc, Ridgefield, CT, USA; 4 Centre Hospitalier Universitaire de Québec – Pav CHUL, Sainte-Foy, Quebec, Canada

## Abstract

**Background:**

The Study of Micardis (telmisartan) in Overweight/Obese patients with Type 2 diabetes and Hypertension (SMOOTH) compared hydrochlorothiazide (HCTZ) plus telmisartan or valsartan fixed-dose combination therapies on early morning blood pressure (BP), using ambulatory BP monitoring (ABPM).

**Methods:**

SMOOTH was a prospective, randomized, open-label, blinded-endpoint, multicentre trial. After a 2- to 4-week, single-blind, placebo run-in period, patients received once-daily telmisartan 80 mg or valsartan 160 mg for 4 weeks, with add-on HCTZ 12.5 mg for 6 weeks (T/HCTZ or V/HCTZ, respectively). At baseline and week 10, ambulatory blood pressure (ABP) was measured every 20 min and hourly means were calculated. The primary endpoint was change from baseline in mean ambulatory systolic and diastolic blood pressure (SBP; DBP) during the last 6 hours of the 24-hour dosing interval.

**Results:**

In total, 840 patients were randomized. At week 10, T/HCTZ provided significantly greater reductions versus V/HCTZ in the last 6 hours mean ABP (differences in favour of T/HCTZ: SBP 3.9 mm Hg, p < 0.0001; DBP 2.0 mm Hg, p = 0.0007). T/HCTZ also produced significantly greater reductions than V/HCTZ in 24-hour mean ABP (differences in favour of T/HCTZ: SBP 3.0 mm Hg, p = 0.0002; DBP 1.6 mm Hg, p = 0.0006) and during the morning, daytime and night-time periods (p < 0.003). Both treatments were well tolerated.

**Conclusion:**

In high-risk, overweight/obese patients with hypertension and type 2 diabetes, T/HCTZ provides significantly greater BP lowering versus V/HCTZ throughout the 24-hour dosing interval, particularly during the hazardous early morning hours.

## Background

Hypertension, obesity and type 2 diabetes are cardiovascular risk factors that commonly occur together. Insufficient suppression of the renin-angiotensin-aldosterone system (RAAS) has been implicated in the development of obesity-related high arterial pressure, and is linked with insulin resistance and type 2 diabetes [1,2]. RAAS blockade may, therefore, be particularly beneficial in the antihypertensive treatment of patients with type 2 diabetes, features of metabolic syndrome and obesity, particularly as this population is poorly controlled. In a cross-sectional prevalence study of 45,125 subjects from Germany, hypertension (blood pressure [BP] ≥ 140/90 mm Hg) was twice as common in obese as in non-obese patients (60.6 vs. 34.3%, respectively) [3]. Furthermore, BP control in diagnosed and treated obese hypertensive patients was extremely low (overall response [OR] = 0.8).

Adequate BP control is also overlooked during the morning hours. During this time there is typically a surge in BP, which is associated with a high incidence of cerebro- and cardiovascular events [4]. Consequently, the early morning hours are an important therapeutic target for antihypertensive treatment [5].

Telmisartan is a once-daily angiotensin II receptor blocker (ARB) with the longest plasma half-life of any ARB, providing 24-hour coverage of BP control from a single daily dose; the angiotensin type 1 (AT_1_) versus AT_2 _receptor affinity ratio for telmisartan is 3000-fold; however, it is higher for valsartan (about 20,000-fold) [6-8]. In two randomized studies of 1,279 hypertensive patients, telmisartan 80 mg significantly reduced the early morning systolic BP (SBP) surge compared with ramipril 10 mg [9]. Another ARB, valsartan has been shown to improve obesity-related disorders, reduce body mass index (BMI) and lower BP [10]. Based on such findings, it is therefore relevant to compare telmisartan with valsartan. A previous pooled analysis of two studies in patients with uncomplicated hypertension showed that telmisartan 80 mg provided SBP reductions in the last 6 hours of the dosing interval and in the 24-hour mean that were superior to the equipotent valsartan 160 mg (by 2.7 and 2.0 mm Hg, respectively) [11]. In addition, two recent studies have shown that telmisartan 80 mg plus hydrochlorothiazide (HCTZ) 25 mg was superior to valsartan 160 mg plus HCTZ 25 mg [12,13]. However, there are few studies comparing telmisartan and valsartan when used in combination with low-dose HCTZ 12.5 mg, and few direct ARB comparisons in obese hypertensive patients with type 2 diabetes.

In this Study of Micardis (telmisartan) in Overweight/Obese patients with Type 2 diabetes and Hypertension (SMOOTH), the effect on early morning BP of the fixed-dose combinations, telmisartan 80 mg plus HCTZ 12.5 mg (T/HCTZ) and valsartan 160 mg plus HCTZ 12.5 mg (V/HCTZ), were compared using ambulatory BP monitoring (ABPM). This is one of the largest ABPM studies performed in obese hypertensive patients.

## Methods

Men and women aged ≥ 30 years with mild-to-moderate hypertension, defined as mean seated cuff SBP 140–179 mm Hg and/or diastolic BP (DBP) 95–109 mm Hg were randomized. Patients were also required to have 24-hour mean ambulatory SBP ≥ 130 mm Hg and/or DBP ≥ 85 mm Hg, type 2 diabetes that had remained stable and controlled for ≥ 3 months, defined as glycolated haemoglobin (HbA_1C_) ≤ 10% and a BMI ≥ 27 kg/m^2 ^in non-Asians and ≥ 24 kg/m^2 ^in Asians (patients' upper arm circumference was required to be ≤ 52 cm to maximize the accuracy of BP readings).

Patients were excluded from randomization if they had mean seated SBP ≥ 180 mm Hg or mean seated DBP ≥ 110 mm Hg during any visit of the placebo run-in period, or fasting serum glucose > 17 mmol/l (300 mg/dl). Premenopausal women who were nursing, pregnant or not using adequate contraception were excluded. In addition, patients with a history of coronary disease, congestive heart failure, or a recent acute cardiovascular event (previous 3 months) or stroke (previous 6 months) were excluded, as were those with secondary hypertension. Patients were not eligible for randomization if they had hepatic or renal impairment. Night-shift workers were also excluded.

The use of corticosteroids, cholestyramine and colestipol resins was prohibited throughout the SMOOTH study. In addition, medications known to affect BP were not allowed. These included vasopressors, vasodilators, beta-agonists and -antagonists, nitroglycerin spray or sublingual tablets, theophylline, dipyridamole, monoamine oxidase inhibitors, phenothiazine and tricyclic antidepressants, chronic use of oral, nasal or topical decongestants and antihypertensive medication other than the study drugs.

### Study design

SMOOTH (clinical trial number: 502.399) used a prospective, randomized, open-label, blinded-endpoint (PROBE), multicentre design. A published meta-analysis has shown that blinded 24-h systolic and diastolic ambulatory BP results from PROBE studies evaluating antihypertensive therapy were statistically equivalent to double-blind, placebo-controlled (DBPC) studies. [14]. These findings show that the PROBE study design is an attractive and validated alternative to the DBPC design, particularly in hypertension trials using ABPM. The PROBE design maintains the strict randomization procedure, incorporates clearly defined (and blinded) endpoints that help eliminate bias, and allows objective comparison of therapies by an independent body.

After a 2- to 4-week, single-blind, placebo run-in period in SMOOTH, patients who met inclusion and exclusion criteria were randomized on a 1:1 basis to 4 weeks' monotherapy with either telmisartan 80 mg or valsartan 160 mg, after which all subjects received add-on HCTZ 12.5 mg for the following 6 weeks. Randomization, stratified by study centre, was by an interactive voice response system and determined using ClinPro/LBL Version 5.2 (Clinical Systems, Inc. software, USA). Patients were instructed to take study medication once daily in the morning with water (and consistently with or without food) at approximately the same time each day (0900 hours ± 1 hour).

The SMOOTH study was conducted at 118 centres in Argentina, Australia, Canada, Mexico, New Zealand, South Korea, Taiwan and the USA, following approval by the relevant local Institutional Review Board or central Independent Ethics Committee, and according to the principles of Good Clinical Practices. SMOOTH is part of the PROTECTION Programme, which is supported by Boehringer Ingelheim.

### Efficacy and safety evaluations

ABPM was conducted at baseline (end of placebo run-in) and week 10 using a Spacelab Model 90207 monitor (OSI Systems Company, Issaquah, WA, USA), which was applied before the patient took the scheduled study medication. ABP was automatically measured every 20 min during the 24-hour monitoring period following dosing, with hourly means calculated relative to both the dosing time and clock time. At each scheduled visit, clinic BP was recorded using a mercury sphygmomanometer, and was defined as the mean of three seated measurements collected 2 min apart, after the patient had been seated quietly for 5 min.

The primary endpoint in the SMOOTH study was the change from baseline in mean ambulatory SBP and DBP during the last 6 hours of the 24-hour dosing interval. Secondary endpoints were changes from baseline in mean 24-hour, morning (0600 hours to noon), daytime (noon to 2200 hours), and night-time (2200 hours to 0600 hours) ambulatory SBP and DBP; changes in trough seated clinic BP; and response rates based on both 24-hour mean ambulatory SBP (24-hour mean SBP < 130 mm Hg and/or reduction ≥ 10 mm Hg) and DBP (24-hour mean DBP < 80 mm Hg and/or reduction ≥ 10 mm Hg). The incidence and severity of adverse events were monitored throughout the study.

### Statistics

For the primary endpoint, assuming standard deviations of 12 and 8 mm Hg for SBP and DBP, a sample size of 337 evaluable patients per treatment group would have had approximately 90% power to detect a treatment difference of 3 mm Hg (SBP) and 2 mm Hg (DBP) at the 5% (two-sided) level of significance.

Statistical analysis was performed on the full analysis set, which included all patients who received a dose of study medication and who had successful ABPMs at baseline and at week 10. Treatment effects were compared by analysis of covariance, with treatment regimen and centre as main effects, and the last 6-hour mean ABP at baseline as covariate.

Testing of multiple endpoints (changes in the last 6-hour mean ambulatory SBP and DBP) utilized a pre-defined hierarchical testing procedure in the following order: (1) comparison of treatment groups based on SBP; and (2) if significant, comparison based on DBP. Thus, all significance testing utilized a two-sided 5% level of significance.

## Results

A total of 840 patients who met inclusion and exclusion criteria were randomized to either T/HCTZ (n = 428) or V/HCTZ (n = 412). Baseline characteristics of randomized patients were comparable for the two treatment groups and are shown in Table 1. About 10% of randomized patients in each treatment group did not complete the trial, most frequently as a result of adverse events (T/HCTZ, n = 10 [2.3%]; V/HCTZ, n = 17 [4.1%]) or withdrawal of consent (T/HCTZ, n = 11 [2.6%]; V/HCTZ, n = 6 [1.5%]). The full analysis set used for the efficacy analyses included 743 patients (T/HCTZ, n = 378; V/HCTZ, n = 365) who had successful ABPM measurements at both their baseline and final visits.

**Table 1:** Patient demographics and baseline characteristics of randomized patients.

	**T/HCTZ (n = 428)**	**V/HCTZ (n = 412)**
Gender (n [%])		
Men	230 (53.7)	239 (58.0)
Women	198 (46.3)	173 (42.0)
Age		
Mean ± SD (years)	58.5 ± 9.7	59.2 ± 9.7
≥ 65 years (n [%])	122 (28.5)	119 (28.9)
Race (n [%])		
White	292 (68.2)	292 (70.9)
Black	61 (14.3)	59 (14.3)
Asian	75 (17.5)	61 (14.8)
BMI		
Mean ± SD (kg/m^2^)	33.6 ± 5.7	33.4 ± 5.3
Hypertension duration		
Mean ± SD (years)	9.2 ± 9.5	8.9 ± 9.2
24-hour ABP		
Mean SBP ± SD (mm Hg)	148.0 ± 11.9	147.8 ± 12.4
Mean DBP ± SD (mm Hg)	83.1 ± 9.2	83.3 ± 10.2
Trough seated BP		
Mean SBP ± SD (mm Hg)	156.8 ± 12.5	157.1 ± 12.3
Mean DBP ± SD (mm Hg)	92.1 ± 9.3	91.7 ± 9.8
HbA1c (%)		
Mean ± SD	7.0 ± 1.1	7.2 ± 1.2

ABP: ambulatory blood pressure; DBP: diastolic blood pressure; T/HCTZ: telmisartan 80 mg plus HCTZ 12.5 mg; V/HCTZ; valsartan 160 mg plus HCTZ 12.5 mg; SBP: systolic blood pressure.

### Reduction of ambulatory blood pressure in the last 6 hours of the 24-hour dosing interval

**Figure 1:**
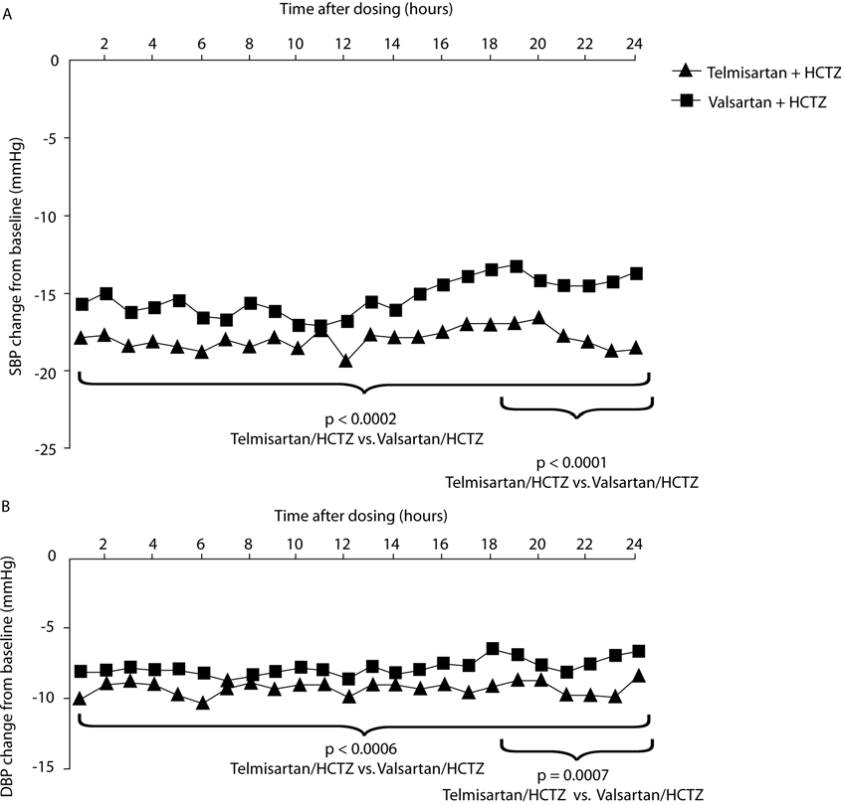
Changes from baseline in hourly means for (A) SBP and (B) DBP after treatment with T/HCTZ or V/HCTZ at 10 weeks. Squares: T/HCTZ; triangles: V/HCTZ.

**Figure 2:**
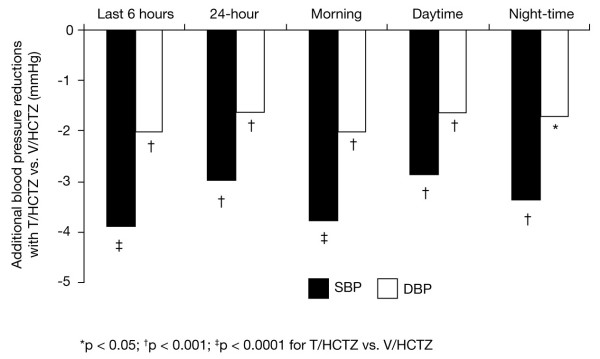
Additional reductions with T/HCTZ compared with V/HCTZ in mean ambulatory SBP and DBP during various periods of the 24-hour dosing interval after treatment for 10 weeks. Black: SBP; white: DBP.

After 10 weeks' treatment, T/HCTZ provided significantly greater mean BP lowering compared with V/HCTZ in the last 6 hours of the 24-hour dosing interval (Figs. 1 and 2). Mean SBP/DBP changes were -18.4/-9.7 mm Hg in T/HCTZ-treated patients and -14.5/-7.7 mm Hg in V/HCTZ-treated patients. This equates to a significant adjusted mean difference in favour of T/HCTZ of 3.9 mm Hg (95% confidence interval [CI] -5.8, -2.1) in SBP (p < 0.0001) and 2.0 mm Hg (95% CI -3.2, -0.8) in DBP (p = 0.0007). Overall, the mean BP was 139.1/82.4 mm Hg (V/HCTZ) and 135.6/81.2 mm Hg (T/HCTZ).

Mean BP reductions in the last 6 hours were not significantly affected by age group (< 65 years vs. ≥ 65 years) or race. Women had significantly greater BP reductions than men; 20.6/11.3 versus 16.8/8.4 mm Hg in patients taking T/HCTZ, and 16.1/9.4 versus 13.4/6.5 mm Hg in those taking V/HCTZ. However, the difference between treatments was consistent in both genders.

### Secondary endpoints

The changes from baseline in hourly means for SBP and DBP after 10 weeks of treatment with either T/HCTZ or V/HCTZ are shown in Figure 1, and the additional reductions with T/HCTZ versus V/HCTZ during various periods are depicted in Figure 2. T/HCTZ produced significantly greater reductions in 24-hour mean BP than V/HCTZ during the 24-hour dosing interval (SBP 3.0 mm Hg, p = 0.002; DBP 1.6 mm Hg, p = 0.0006). In addition, during the morning, daytime and night-time periods, patients receiving T/HCTZ had significantly greater reductions in mean ambulatory SBP and DBP compared with those taking V/HCTZ (p < 0.003) (Fig. 2). Ambulatory SBP response rates were high in patients taking T/HCTZ (79.9%) versus V/HCTZ (75.6%). Ambulatory DBP response rates were similarly high in patients taking T/HCTZ (79.9%) versus V/HCTZ (77.3%). Clinic DBP and SBP response rates were also high in patients taking T/HCTZ (80.6% and 82.3%, respectively) versus V/HCTZ (75.5% and 75.0%). Clinic BP control (< 130/80 mm Hg) was higher in patients taking T/HCTZ than V/HCTZ (26.4% vs. 24.0%, respectively). Clinic DBP control was also higher in the T/HCTZ group than V/HCTZ group (77.4% vs. 71.9%, respectively).

Reductions in trough seated clinic BP after 10 weeks' treatment were significantly greater with T/HCTZ compared with V/HCTZ (SBP 3.2 mm Hg, p = 0.0017; DBP 1.2 mm Hg; p = 0.0446). The advantage of telmisartan was already apparent after 4 weeks of treatment with monotherapy (SBP 2.5 mm Hg: p = 0.0106; DBP 0.8 mm Hg: p = 0.1370). The mean BP achieved with monotherapy was 146.6/86.3 mm Hg (valsartan) and 143.3/85.5 mm Hg (telmisartan). Clinic BP control (< 130/80 mm Hg) was also higher following monotherapy with telmisartan compared with valsartan (13.6 vs. 10.6%, respectively) although these values were still low. Clinic DBP control was, however, much higher following monotherapy (64.5% [telmisartan] and 63.1% [valsartan]).

### Safety

The overall incidence of adverse events was low with at least one adverse event reported by 158 T/HCTZ-randomized patients (36.9%) and 151 (36.7%) V/HCTZ-randomized patients. With the exception of dizziness, which was reported in 14(3.3%) T/HCTZ-randomized patients and one (0.2%) V/HCTZ-randomized patient, there were no differences in the incidence of specific adverse events between treatment groups.

Events were generally mild or moderate in intensity. Serious adverse events were reported by three (0.7%) patients in each treatment group during monotherapy, and four (1.0%) patients (T/HCTZ) and five (1.3%) patients (V/HCTZ) during combination therapy. Adverse events reported as serious were cardiac disorders (acute coronary syndrome, unstable angina, coronary artery disease and myocardial ischaemia), gastro-oesophageal reflux disease, general disorders (asthenia, chest pain, cellulites), injury (ankle fracture and joint dislocation), drug toxicity, increased blood creatine phosphokinase, inadequately controlled diabetes mellitus, athralgia, back pain, nervous system disorders (carotid artery stenosis, cerebral haemorrhage, cerebral thrombosis, headache and intracranial aneurysm), bipolar I disorder and vascular disorders (hypertensive crisis and hypotension).

Treatment-related adverse events were infrequent in the T/HCTZ (27, 6.3%) and V/HCTZ (22, 5.3%) treatment arms; the most common were oedema (0 vs. 4 [1.0%]), dizziness (5 [1.2%] vs. 0), and headache (4 [0.9%] vs. 6 [1.5%] for T/HCTZ and V/HCTZ, respectively). Laboratory markers at baseline and following T/HCTZ or V/HCTZ treatment are summarized in Table 2. The addition of HCTZ did not result in any significant metabolic disturbances.

**Table 2:** Summary of metabolic markers at baseline and on the last day of combination therapy.

	**T/HCTZ**		**V/HCTZ**		Difference* (T/HCTZ – V/HCTZ)
Endpoint, mean ± SD	**Baseline**	**Last day**	**Baseline**	**Last day**	**p value**

Serum triglyceride (mg/dL)	201.7 ± 247	211.8 ± 172	196.3 ± 151	210.5 ± 177	0.91
LDL-C (mg/dL)	165.6 ± 35.4	169.8 ± 38.1	162.8 ± 33.9	165.3 ± 36.0	0.17
HDL-C (mg/dL)	37.1 ± 6.5	36.6 ± 6.3	37.6 ± 7.2	37.1 ± 7.0	0.64
Total cholesterol (mg/dL)	195.7 ± 23.3	198.5 ± 21.7	194.5 ± 19.8	196.3 ± 20.9	0.20
Potassium (mEq/L)	4.4 ± 0.4	4.5 ± 0.4	4.4 ± 0.4	4.4 ± 0.4	0.03
Glucose (mg/dL)	128.8 ± 44.1	138.8 ± 53.1	134.1 ± 49.2	140.4 ± 55.2	0.58
HbA_1c _(%)	6.5 ± 0.3	6.6 ± 0.3	6.6 ± 0.3	6.7 ± 0.4	0.93
Albumin:creatinine ratio	97.6 ± 330	65.1 ± 266	66.7 ± 239	45.1 ± 177	0.97

T/HCTZ: telmisartan 80 mg plus HCTZ 12.5 mg; V/HCTZ: valsartan 160 mg plus HCTZ 12.5 mg; LDL-C: low-density lipoprotein cholesterol; HDL-C: high-density lipoprotein cholesterol; HbA_1c_: glycolated haemoglobin.

*Analysis of change from baseline

## Discussion

In a large, multicentre, PROBE study of overweight/obese patients with hypertension and type 2 diabetes, once-daily T/HCTZ was significantly more effective at lowering BP than V/HCTZ during the last 6 hours of the 24-hour dosing interval. T/HCTZ also produced significantly greater BP reductions than V/HCTZ over the entire 24 hours. Both ARBs were well tolerated.

Patients enrolled in the SMOOTH study were at very high risk of cardiovascular events and stroke because of the presence of additive cardiovascular risk factors. In patients with hypertension, antihypertensive agents are recommended with a BP target of 140/90 mm Hg in patients without diabetes and 130/80 mm Hg in those with diabetes [15,16]. However, there are no specific recommendations for patients with coexisting hypertension, obesity and type 2 diabetes.

Increased activity of the RAAS is associated with obesity-linked hypertension and also with the development of type 2 diabetes. Antihypertensive agents that block the RAAS may be beneficial in patients with type 2 diabetes and features of metabolic syndrome [2]. However, there are few studies focusing on angiotensin blockade in the management of the obese hypertensive patient. ARBs and angiotensin-converting enzyme inhibitors (ACEis) may have favourable metabolic effects in addition to their antihypertensive properties that could be beneficial in these obese patients [2]. This has already been shown with valsartan and irbesartan, both in combination with HCTZ [17,18]. In another study, the Candesartan Role on Obesity and on Sympathetic System (CROSS), candesartan was found to have comparable BP lowering to HCTZ, but the ARB also had a significant benefit on insulin sensitivity [19]. The current study is novel, in that it is one of the first to directly compare ARBs in such a large obese-hypertensive population, and to monitor antihypertensive effectiveness using 24-hour ABPM.

Furthermore, our study further confirms the superiority of telmisartan versus valsartan, when both are used in fixed-dose combination with HCTZ. Although physicians are likely to use an up-titration approach to patient management, there is now evidence to suggest that a fixed-dose combination is beneficial in obese hypertensive patients, and may help to overcome the barrier of poor patient compliance as a result of polypharmacy or lack of efficacy [20]. In the current study, the mean BP achieved with monotherapy was 146.6/86.3 mm Hg (valsartan) and 143.3/85.5 mm Hg (telmisartan), which was improved to 139.1/82.4 mm Hg (valsartan) and 135.6/81.2 mm Hg (telmisartan) when the patients were switched to low-dose combination with T/HCTZ. The percentage of patients achieving target BP (< 130/80 mm Hg) doubled when patients were switched to combination therapy, from 13.6% (telmisartan) to 26.4% (T/HCTZ) and from 10.6% (valsartan) to 24.0% (V/HCTZ).

Acute cardiovascular events and stroke show a strong circadian pattern. The highest incidence of events occurs in the early morning hours, during which there is typically a surge in BP [9]. Unfortunately, this vulnerable time period coincides with a decline in efficacy of most antihypertensive agents because they are relatively short acting. Differential effects between antihypertensive agents on the magnitude of reduction in the early morning hours have been shown in this and previous studies. Telmisartan has shown superiority in the early morning hours over several other antihypertensives, such as ramipril [21-23], losartan [24,25], and valsartan [9]. In two large studies, telmisartan 80 mg changed the overall mean systolic early morning surge by -1.5 mm Hg compared with a change of + 0.3 mmHg by the ACEi ramipril (p = 0.0049) [6]. In the ATHOS study, telmisartan/HCTZ reduced 24-hour SBP to a greater extent than amlodipine/HCTZ [26]. ATHOS showed that antihypertensive agents with a long half-life, such as telmisartan, can maintain efficacy throughout the 24-hour dosing period, thereby providing greater reductions in early morning BP than short-acting agents [6,27]. The improved early morning hypertension control with T/HCTZ in the SMOOTH study may, therefore, be explained by the 24-hour plasma elimination half-life of telmisartan [28]. In contrast, valsartan has a shorter half-life of about 16 hours [6].

BP is strongly correlated with vascular (and overall) mortality without any evidence of a threshold down to at least 115/75 mm Hg. This was demonstrated in a meta-analysis of data from 61 prospective observational studies of patients with vascular disease [29]. In the present study, T/HCTZ provided significant additional reductions versus V/HCTZ of 3.9 mm Hg in SBP and 2.0 in DBP during the last 6 hours of the 24-hour dosing interval. Reductions of this magnitude may be clinically meaningful. For example, in the above meta-analysis, it was calculated that a reduction in SBP of 2 mm Hg was associated with a 7% reduction in risk of mortality from ischaemic heart disease and a 10% reduction in risk of mortality from stroke [29]. In another study, reduction of early morning BP in patients with type 2 diabetes, hypertension, and nephropathy was associated with a significant slowing of the progression of renal damage [30].

In conclusion, in overweight/obese patients with hypertension and type 2 diabetes, once-daily T/HCTZ was significantly more effective than V/HCTZ during the last 6 hours of the 24-hour dosing interval, the time when most cardiovascular events occur [31]. These findings indicate significant potential treatment benefit of T/HCTZ for this at-risk patient population. With strong evidence available directly correlating BP reductions to cardiovascular and cerebrovascular risk reduction, the additional efficacy afforded by T/HCTZ over V/HCTZ may confer a clinical advantage.
